# Health-related physical fitness in children among five Mediterranean countries: a cross-cultural study from the DELICIOUS project

**DOI:** 10.3389/fpubh.2024.1520096

**Published:** 2025-01-07

**Authors:** Mohamed Aly, Noha El-Gyar, Amira M. Shalaby, Osama Abdelkarim

**Affiliations:** ^1^Faculty of Physical Education, Assiut University, Asyut, Egypt; ^2^Department of Pediatric, Faculty of Medicine, Assiut University, Assiut, Egypt

**Keywords:** cross-cultural differences, physical fitness, Mediterranean countries, DELICIOUS project, children

## Abstract

**Background:**

Health-related fitness (HRF) components are essential for supporting healthy growth and reducing long-term health risks in children. This study explored cross-cultural variations in HRF among children from five Mediterranean countries—Egypt, Italy, Lebanon, Portugal, and Spain—within the framework of the DELICIOUS project.

**Methods:**

A total of 860 children participated in the study, including 204 from Egypt (*n* = 204, 11.72 ± 1.46 years), 150 from Italy (9.66 ± 1.10 years), 200 from Lebanon (10.73 ± 1.90 years), 181 from Portugal (11.04 ± 1.83 years), and 125 from Spain (12.33 ± 2.27 years). Participants completed the International Physical Performance Test Profile (IPPTP), which assesses sprint speed (20 m dash), coordination (jumping sideways), upper body strength (push-ups), abdominal strength (sit-ups), lower body power (standing long jump), and cardiovascular endurance (6-min run). Children were categorized into two age groups: 8–10 and 11–14 years. ANCOVA, adjusting for BMI, was performed to analyze differences across countries and age groups.

**Results:**

Analysis revealed significant differences in HRF across countries and age groups (*p* < 0.05). Spanish boys and girls consistently demonstrated superior sprint performance (20 m dash) and cardiovascular endurance (6-min run) compared to peers from other countries. Lebanese and Spanish girls exhibited stronger abdominal performance (sit-ups) than Egyptian girls, while Spanish girls also excelled in lower-body power (standing long jump). These findings underscore cross-cultural variations in HRF outcomes among Mediterranean children.

**Conclusion:**

Cross-cultural differences in physical education programs and sports participation appear to influence HRF in children across the Mediterranean region. These findings underscore the need for culturally tailored physical education strategies and public health initiatives to ensure balanced fitness development in diverse cultural populations.

## 1 Introduction

Physical inactivity is a significant global health challenge, contributing to the prevalence of chronic diseases such as obesity, cardiovascular disease, and diabetes. It is a leading cause of premature mortality, ranking as the fourth-highest cause of death worldwide (1). In 2013, inadequate physical activity (PA) was estimated to have cost the global healthcare system USD 53.8 billion, with substantial expenditures in Europe (USD 11.7 billion) and North America [USD 25.7 billion; (2)]. These economic implications highlight the urgent need for public health initiatives that promote and sustain PA levels, particularly among children and adolescents (3). Beyond its physical health benefits, PA plays a crucial role in brain health and cognitive function (4, 5). Regular activity enhances memory, attention, and executive functioning, while also supporting academic performance by improving concentration, problem-solving abilities, and emotional regulation (6–10). These cognitive and academic advantages underscore the importance of embedding physical activity into children’s daily routines to foster holistic development. While the global burden of physical inactivity is well-established, less attention has been given to how cultural and environmental factors that shape children’s physical activity behaviors and fitness levels across different regions. Understanding these contextual influences is vital for designing targeted strategies to address physical inactivity and its associated challenges worldwide.

Physical education (PE) programs vary significantly across Mediterranean countries, influenced by cultural priorities, educational policies, and the availability of resources for physical activity. In Portugal, PE is mandatory throughout primary and secondary education, with a focus on diverse sports and activities designed to encourage lifelong physical activity (11, 12). Similarly, Spain integrates a comprehensive PE curriculum with culturally prominent sports like soccer, fostering children’s endurance and agility development (13, 14).

Italy includes PE as a core component of the standard school curriculum but emphasizes general fitness and motor skills over competitive sports, prioritizing holistic well-being and overall health rather than intense sports training (15). In Lebanon, while PE is mandatory, the quality and resources available for PE programs vary widely. Participation in physical activity among Lebanese children and youth tends to be low, and PE programs typically emphasize basic physical activity rather than specialized sports training (16, 17). Egypt, in contrast, faces challenges in delivering consistent PE curricula due to resource constraints. Many schools provide limited structured physical activity opportunities, resulting in less emphasis on physical fitness development (18, 19). These disparities in PE priorities, structure, and resources across the five countries likely influence children’s health-related physical fitness (HPF). Research has shown that well-resourced and structured PE programs are strongly associated with improved fitness outcomes (20), highlighting the importance of investing in and standardizing PE initiatives to support children’s physical health and development.

HPF encompasses a set of physical attributes that are essential for overall health and well-being, including cardiorespiratory endurance, muscular strength, muscular endurance, flexibility, and body composition (21). Unlike skill-related fitness, which emphasizes motor skills or athletic performance, HPF focuses on factors that help prevent disease and promote long-term health. Developing strong HPF during childhood not only supports healthy physical growth but also fosters activity patterns that are likely to continue into adolescence and adulthood, reducing the risk of chronic diseases later in life (22).

However, despite its vital role, recent studies have revealed a global decline in HPF among children, raising serious concerns about its potential long-term health impacts (23). Understanding these trends is particularly important in regions where physical fitness outcomes are shaped by unique cultural, environmental, and socio-economic factors. Addressing the decline in HPF requires targeted strategies that consider these contextual influences to promote healthier lifestyles and reduce future health risks.

HRF in children varies widely across regions, influenced by cultural, environmental, and social factors that shape physical activity levels and fitness outcomes (24–26). The Social Ecological Model offers a valuable framework for understanding these influences, emphasizing that individual health behaviors are shaped by multiple layers of interaction, including societal norms, family dynamics, and educational environments (3). Complementing this, the Life Course Theory highlights how early-life experiences—such as access to physical education (PE) and sports—can significantly influence health trajectories into adulthood (22).

Although research comparing children’s HRF across countries is limited, existing studies underscore the importance of cultural contexts in fitness outcomes. For instance, Luz et al. (27), compared motor competence and HRF between children from Portugal and the United States, while Bardid et al. (28) examined motor competence differences between children in Australia and Belgium and revealed important cultural differences. In the latter study, Belgian children outperformed their Australian peers in motor tasks such as jumping sideways and hopping for height, a disparity linked to cultural factors such as PE curricula and active transportation practices. These cross-cultural studies highlight how specific motor skills are nurtured differently depending on cultural priorities and contexts. Standardized fitness assessments can uncover these variations, providing insights into the cultural influences that shape skill development. Understanding HRF within diverse cultural settings is critical for designing equitable public health strategies that enhance fitness opportunities for children worldwide (29).

The Mediterranean region offers a unique opportunity for cross-cultural comparisons of HPF due to its blend of shared cultural traits—such as the prevalence of the Mediterranean diet (30, 63)—and distinct variations in physical education (PE) practices and youth sports cultures. For instance, while children in Spain and Portugal may share similar dietary habits, their approaches to sports participation differ. Portuguese children often specialize in a single sport, such as soccer, whereas their peers in other countries are more likely to engage in a broader range of activities (31, 32). In contrast, children in Egypt may face different opportunities for physical activity due to disparities in school-based PE programs and the availability of community sports initiatives (33). Despite these acknowledged cultural differences, limited research exists on how these factors shape physical fitness outcomes among children within the Mediterranean context. Such studies are critical for understanding the interplay between cultural practices and fitness development, paving the way for tailored interventions to promote equitable health and fitness across the region.

This study focuses on five Mediterranean countries—Egypt, Italy, Lebanon, Portugal, and Spain—examining HPF across two age groups (8–10 and 11–14 years). It aims to explore how cultural and environmental factors influence the development of HPF in youth, with a particular focus on identifying differences in fitness levels among children in these countries. The findings are expected to offer valuable insights for shaping public health strategies and refining physical education (PE) practices, ultimately supporting improved fitness outcomes in diverse cultural contexts.

## 2 Materials and methods

### 2.1 Participants

The study sample was drawn from partner countries within the DELICIOUS consortium: Egypt, Italy, Lebanon, Portugal, and Spain. primary goals and objectives of the DELICIOUS project, a comprehensive descriptive and intervention study aimed at promoting the Mediterranean diet and an active lifestyle among school children and adolescents, have been detailed elsewhere (30, 64). For this study, a total of 860 children aged 8–14 years were recruited from participating schools in urban areas across five Mediterranean cities: Assiut (Egypt), Lisbon (Portugal), Beirut (Lebanon), Cordoba (Spain), and Giugliano in Campania (Italy). To account for potential age-related differences in fitness levels, participants were stratified into two age groups: 8–10 years and 11–14 years. Written informed consent was obtained from parents or legal guardians of all participants prior to the children’s participation. The study adhered to the ethical standards outlined in the Declaration of Helsinki and was approved by the ethics committee of Mondragon University (no. IEB-20230704).

### 2.2 Measures

#### 2.2.1 Physical fitness

The International Physical Performance Test Profile (IPPTP) is a standardized and validated test battery designed to assess HPF in children and adolescents. It provides a comprehensive evaluation of key fitness components, including speed, agility, strength, endurance, and coordination, making it particularly well-suited for cross-cultural studies (6, 18, 34–37). Developed based on the methodologies of Bös and Mechling (38) and the German Motor Test 6–18 (39), the IPPTP comprises eight test items that encompass the five fundamental dimensions of physical fitness: endurance, strength, speed, coordination, and flexibility. For this study, six specific tests from the IPPTP were selected to target the core aspects of physical fitness relevant to children’s HPF. These tests were chosen for their high validity in measuring fitness components across culturally diverse populations and their robustness in cross-cultural applications. For this study, six tests were selected to target core aspects of children’s HPF: the 20 m Dash Test assessed speed by recording sprint times over 20 meters; the Sideways Jumping Test measured agility and coordination through the number of lateral jumps in 15 s; the Push-Up Test evaluated upper body strength and endurance by counting the total push-ups completed in 40 s; the Sit-Up Test assessed core strength through the number of sit-ups performed in 40 s; the Standing Long Jump Test measured lower body strength by the distance jumped from a standing position in centimeters; and the 6-Minute Run assessed cardiovascular endurance by the total distance covered in meters. All tests were conducted under standardized conditions to ensure consistency and reliability across the diverse sample populations. The reproducibility and validity of the IPPTP have been established in previous research (6, 18). For additional details on these test items, readers are referred to the corresponding manuals (35, 39).

#### 2.2.2 Anthropometry

Height and weight were recorded prior to testing. Height was measured to the nearest 0.1 cm using a portable stadiometer (Seca 213, Seca GmbH & Co. KG, Hamburg, Germany), while weight was measured using a Tanita digital body composition scale (BF-350 Total Body Composition Analyzer, Amsterdam, the Netherlands), following standardized anthropometric protocols. Body mass index (BMI) was then calculated using the formula: BMI = weight (kg)/height (m^2^).

### 2.3 Statistical analysis

Descriptive statistics (means and standard deviations) were computed to characterize HPF across age groups and sex. Prior to conducting further analyses, data were assessed for normality using the Shapiro–Wilk test and for homogeneity of variances using Levene’s test to ensure the assumptions required for parametric testing were met. To account for the potential influence of body mass index (BMI), analyses were adjusted for BMI as a covariate. Differences in physical fitness measures across countries (Egypt, Italy, Lebanon, Portugal, and Spain) and age groups (8–10 years and 11–14 years) were examined separately for boys and girls by a two-way analysis of covariance (ANCOVA) was conducted. *Post hoc* pairwise comparisons were conducted using the Games-Howell test, which is robust to unequal variances and sample sizes. Significance was set at *p* < 0.05 for all analyses. Data were analyzed using R software (40), with *post hoc* tests performed using the “PMCMRplus” package. Effect sizes (eta squared) were calculated to evaluate the magnitude of differences between groups.

## 3 Results

### 3.1 Sample characteristics

Table 1 presents descriptive statistics for height, weight, BMI, and HPF variables across five Mediterranean countries, stratified by age group and sex. For both sexes, ANCOVA revealed significant performance improvements across age groups (*p* < 0. 01) for all fitness variables, except for push-ups, sit-ups, standing long jump, and the 6-min run in boys (Table 2).

**Table 1:** Health-related fitness components (mean ± SD) by age group and sex across five Mediterranean countries.

Variable	8–10 years					11–14 years				
	Egypt	Italy	Lebanon	Portugal	Spain	Egypt	Italy	Lebanon	Portugal	Spain
Boys										
Height (cm)	139.26 ± 6.76	140.58 ± 7.41	136.93 ± 8.34	134.89 ± 5.48	146.83 ± 8.07	153.55 ± 10.88	144.09 ± 7.21	158.13 ± 9.04	155.05 ± 9.52	162.40 ± 12.57
Weight (kg)	33.96 ± 7.89	39.40 ± 11.09	33.39 ± 9.67	31.35 ± 6.05	45.06 ± 11.53	45.81 ± 12.50	40.61 ± 10.21	50.42 ± 12.42	45.05 ± 9.77	56.47 ± 14.61
BMI (kg/m^2^)	17.40 ± 3.27	19.77 ± 4.37	17.52 ± 3.35	17.14 ± 2.52	20.85 ± 4.64	19.20 ± 3.98	19.31 ± 3.34	20.02 ± 4.02	18.58 ± 2.84	21.35 ± 5.03
20 m dash	4.38 ± 0.39	4.55 ± 0.36	4.59 ± 0.46	4.39 ± 0.40	4.32 ± 0.42	3.97 ± 0.47	4.03 ± 0.28	4.01 ± 0.40	4.06 ± 0.62	3.88 ± 0.58
JSW (n)	22.26 ± 3.54	29.07 ± 4.52	23.02 ± 7.75	31.76 ± 6.90	34.33 ± 6.76	25.60 ± 4.42	33.30 ± 8.10	24.64 ± 4.20	35.80 ± 5.31	34.68 ± 5.96
PU (n)	13.13 ± 2.87	16.14 ± 4.21	15.14 ± 4.23	14.89 ± 4.10	14.17 ± 4.77	14.27 ± 3.63	18.13 ± 6.94	15.75 ± 3.79	18.00 ± 5.06	16.74 ± 6.83
SU (n)	19.00 ± 3.85	21.72 ± 4.05	20.34 ± 6.47	22.49 ± 5.67	21.72 ± 2.30	19.96 ± 5.21	25.09 ± 7.13	25.30 ± 5.69	26.35 ± 6.41	27.85 ± 6.80
SLJ (cm)	112.13 ± 21.85	134.51 ± 14.71	135.43 ± 20.80	136.97 ± 16.17	132.39 ± 14.16	135.24 ± 25.38	137.83 ± 11.26	150.32 ± 28.83	164.95 ± 29.73	149.06 ± 31.38
6 min run	759.04 ± 117.89	897.26 ± 159.34	907.52 ± 140.12	1015.62 ± 77.96	996.61 ± 171.68	866.97 ± 117.54	991.91 ± 227.70	964.02 ± 160.22	1100.23 ± 196.92	1093.70 ± 211.18
Girls										
Height (cm)	143.08 ± 7.63	140.12 ± 7.93	138.50 ± 7.19	137.39 ± 9.30	146.21 ± 7.54	151.82 ± 9.86	142.94 ± 7.34	156.96 ± 8.57	155.33 ± 8.65	156.59 ± 8.39
Weight (kg)	39.50 ± 10.12	38.49 ± 8.85	34.21 ± 8.60	33.24 ± 7.25	43.00 ± 9.52	46.18 ± 10.95	41.63 ± 8.74	48.76 ± 11.09	44.88 ± 9.69	51.02 ± 11.21
BMI (kg/m^2^)	19.16 ± 4.09	19.42 ± 3.09	17.72 ± 3.78	17.43 ± 2.43	19.95 ± 3.46	19.89 ± 3.73	20.49 ± 4.89	19.68 ± 3.61	18.47 ± 2.98	20.67 ± 3.64
20 m dash	4.95 ± 0.50	4.76 ± 0.48	4.89 ± 0.63	4.55 ± 0.38	4.30 ± 0.26	4.57 ± 0.49	4.25 ± 0.43	4.11 ± 0.36	4.35 ± 0.56	4.22 ± 0.42
JSW (n)	18.23 ± 3.06	27.81 ± 3.96	20.58 ± 6.27	29.38 ± 3.44	31.21 ± 6.52	21.80 ± 3.06	30.25 ± 5.69	23.77 ± 4.42	31.82 ± 4.76	33.35 ± 6.86
PU (n)	8.31 ± 2.71	14.49 ± 2.61	13.37 ± 4.21	13.73 ± 2.80	10.93 ± 3.52	10.02 ± 2.97	16.06 ± 3.38	14.45 ± 2.93	15.71 ± 4.05	13.76 ± 4.01
SU (n)	9.85 ± 4.63	19.69 ± 3.65	15.79 ± 4.71	19.61 ± 4.00	18.50 ± 4.64	11.36 ± 4.27	22.50 ± 4.73	22.29 ± 5.90	23.18 ± 3.92	21.72 ± 6.75
SLJ (cm)	88.96 ± 13.99	128.01 ± 14.50	121.87 ± 15.88	131.79 ± 11.23	119.00 ± 13.05	101.03 ± 17.23	131.19 ± 14.04	138.00 ± 26.15	158.06 ± 20.39	125.41 ± 27.35
6 min run	691.88 ± 75.12	810.62 ± 152.56	836.10 ± 105.32	945.00 ± 99.66	902.00 ± 179.40	723.12 ± 62.40	870.94 ± 71.83	854.21 ± 111.38	992.61 ± 155.67	913.09 ± 162.78

JSW, jumbling sideways; PU, push up; SU, set up; SLJ, standing long jump.

**Table 2:** Interaction and main effects on health-related fitness variables according to country and age group.

Variable	*F*_Country_	*η_p_^2^*	*F*_age_	*η_p_^2^*	*F*_Country × age_	*η_p_^2^*
Boys						
20 m dash (sec)	4.59**	0.04	9.98**	0.02	1.59	0.01
JSW (*n*)	0.57	0.0053	15.78**	0.04	1.64	0.02
PU (*n*)	1.93	<0.001	0.01	<0.001	0.29	<0.001
SU (*n*)	0.79	0.0073	3.32	0.0077	2.65*	0.02
SLJ (cm)	3.03**	0.03	2.35	0.0054	3.85**	0.03
6 min run (m)	2.95*	0.03	3.67	0.0085	1.95	0.02
Girls						
20 m dash (sec)	9.58**	0.09	14.85**	0.03	2.08	0.02
JSW (*n*)	1.90	0.02	9.95**	0.02	1.98	0.02
PU (*n*)	1.80	0.02	6.56*	0.02	0.28	0.002
SU (*n*)	5.42**	0.05	11.15**	0.03	0.41	0.0039
SLJ (cm)	2.56*	0.02	7.23**	0.02	2.08	0.02
6 min run (m)	2.21	0.02	8.69**	0.02	2.04	0.02

*p < 0.05, **p < 0.01. JSW, jumbling sideways; PU push up; SU, set up; SLJ, standing long jump.

### 3.2 Cross-cultural differences in HRP components

**Figure 1:**
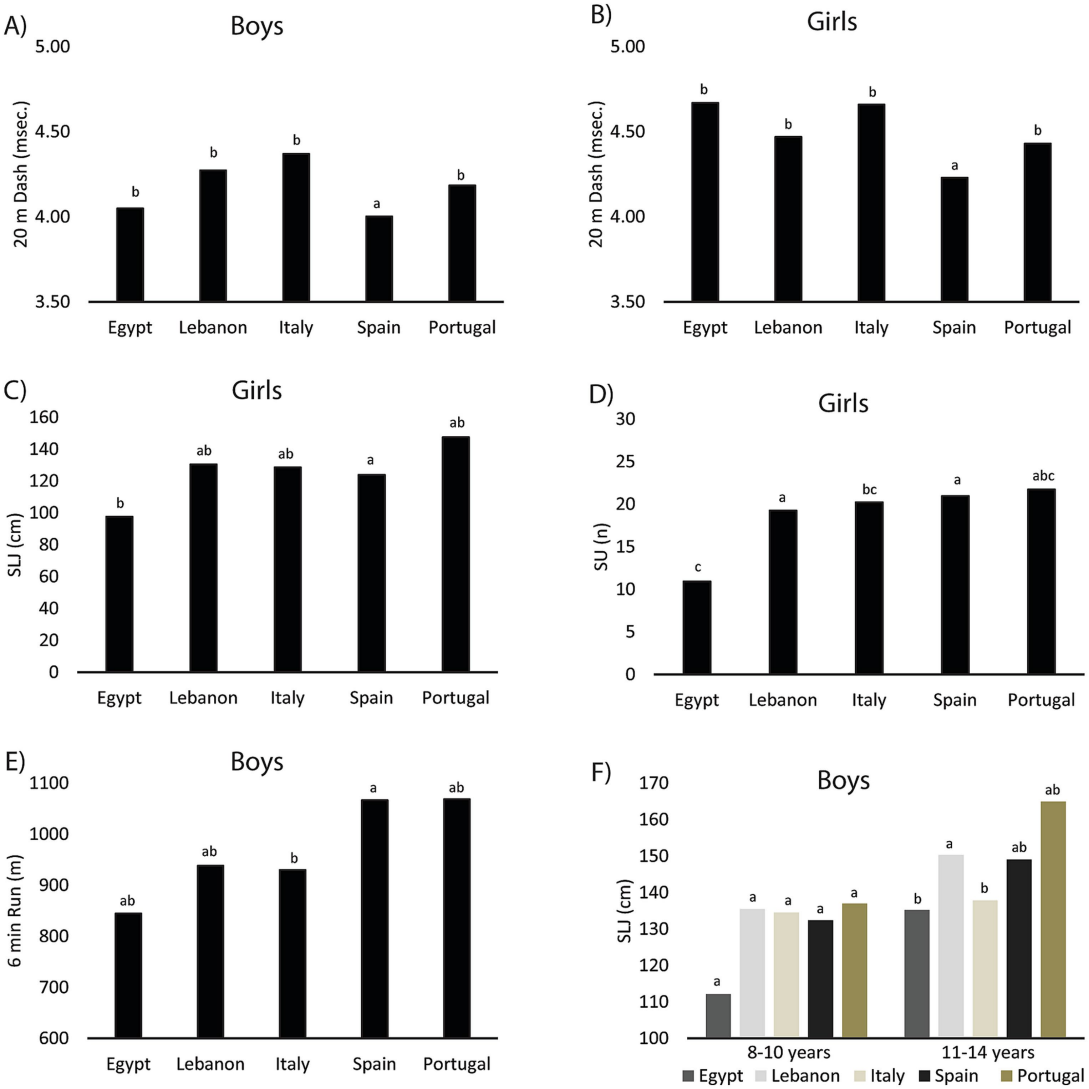
Performance values for boys and girls from Egypt, Italy, Lebanon, Portugal, and Spain in the following tests: **(A,B)** 20 m Dash, **(C,F)** Standing Long Jump (SLJ), **(D)** Sit-Up (SU), and **(E)** 6-Minute Run. Countries with the same letters are not significantly different.

The analysis of ANCOVA, controlling for BMI and examining country and age as factors, revealed significant cross-cultural differences in HRP components. For the 20 m dash, Spanish boys and girls demonstrated significantly faster sprint times than participants from Egypt, Lebanon, Italy, and Portugal, who showed no differences among themselves (Figures 1A,B). In the standing long jump, Egyptian girls exhibited significantly lower performance compared to Spanish girls, while participants from Lebanon, Italy, and Portugal did not differ significantly from either group (Figure 1C). The sit-up test showed that Lebanese and Spanish girls performed significantly better than Egyptian girls, with no significant differences between girls from Italy, Portugal, and Egypt (Figure 1D). Lastly, for the 6-min run, Spanish boys outperformed Italian boys, while participants from Egypt, Lebanon, and Portugal showed no significant differences compared to either Spain or Italy (Figure 1E). These findings highlight notable cultural variations in specific physical fitness components across the sampled countries.

### 3.3 Country × age group interaction effects

For the standing long jump test in boys, ANCOVA revealed a significant interaction effect between country and age group. Among the younger age group (8–10 years), no significant differences were observed between the five counties. In the older age group (11–14 years), boys from Portugal exhibited the best performance, and boys from Lebanon performed significantly better than those from Egypt and Italy, with no significant differences observed among boys from Portugal, Spain, and Lebanon (Figure 1F). For the sit-up test in boys, a significant interaction effect between country and age group was observed, but *post hoc* comparisons did not reveal any significant pairwise differences (*p* > 0.05).

## 4 Discussion

This study assessed HPF levels among children from five Mediterranean countries—Egypt, Italy, Lebanon, Portugal, and Spain—while accounting for BMI as a covariate to explore how cultural and environmental factors influence fitness development. Significant differences were observed across countries and age groups in all fitness components, even after adjusting for BMI. Spain and Portugal consistently outperformed other countries in endurance, coordination, and strength tests, while Egypt demonstrated lower performance across several areas. These findings emphasize the influence of structured physical education (PE) programs and sports participation on fitness outcomes, underlining the need for tailored public health policies in countries with lower fitness levels to improve HPF outcomes (1).

Adjusting for BMI revealed that cross-country differences in HPF persisted across fitness components. The present study showed that boys and girls from Spain exhibited significantly faster sprint times compared to participants from Egypt, Lebanon, Italy, and Portugal, with no significant differences among the latter group, highlighting the potential influence of Spain’s structured PE programs and sports culture on sprint performance. Spain’s emphasis on endurance-based and speed-focused sports, such as soccer and track and field, likely contributes to the development of sprint-specific motor skills and physical conditioning from an early age (41). In contrast, the lack of significant differences among Egypt, Lebanon, Italy, and Portugal may reflect a more generalist approach to PE curricula (42) or a lack of targeted sprint training in these countries (43–45). Additionally, Spain’s investment in youth sports infrastructure and participation in organized sports might create opportunities for regular practice, which enhances speed and power output in sprinting tasks (46, 47). These results underscore the role of cultural and environmental factors in shaping specific fitness components, such as sprint speed, and highlight the need for other countries to consider incorporating more sprint-focused activities into their PE programs to enhance athletic. Importantly, BMI did not substantially alter these patterns, indicating that cultural and environmental factors may play a stronger role than body composition in shaping HPF.

In the sit-up test, girls from Lebanon and Spain significantly outperformed their peers from Egypt, while participants from Italy and Portugal showed no significant differences compared to Egyptian girls. This highlights cross-cultural variations in abdominal strength and endurance, which may be influenced by differences in PE program design. Lebanon’s sporadic but targeted focus on fitness components such as core strength (48), and Spain’s comprehensive approach to physical training, appears to foster better performance in these areas (49). On the other hand, cultural and infrastructural challenges in Egypt may limit children’s opportunities to engage in activities that build abdominal strength, particularly for girls (19).

The standing long jump results revealed that girls from Egypt performed significantly worse compared to those from Spain, while participants from Lebanon, Italy, and Portugal demonstrated comparable performance, with no significant differences from either Egypt or Spain. These findings highlight disparities in lower-body power across countries, likely reflecting differences in the emphasis on explosive strength activities in PE programs. For boys, the significant interaction effect between country and age group for the standing long jump test highlights important cross-cultural and developmental variations in physical fitness. The lack of significant differences among countries in the younger age group (8–10 years) suggests that early development in lower-body strength and explosive power, as measured by the standing long jump, may be relatively homogeneous across the sampled populations. However, in the older age group (11–14 years), boys from Portugal and Lebanon demonstrated superior performance, with Portuguese boys leading, and Lebanese boys significantly outperforming their peers from Egypt and Italy. This finding may reflect differences in cultural and environmental factors that influence physical activity opportunities and training habits in these countries, underscoring the potential benefits of targeted physical education interventions during critical developmental periods (50).

The 6-min run results highlighted Spain’s consistent advantage in cardiovascular endurance, with Spanish boys outperforming their Italian counterparts significantly. Participants from Egypt, Lebanon, and Portugal demonstrated similar performance levels, suggesting comparable cardiovascular endurance across these countries. Spain’s superior performance could be attributed to a stronger emphasis on endurance-based sports and structured aerobic activities within its physical education curricula, which likely contribute to better cardiovascular fitness. The significant interaction effect for the sit-up test in boys, despite the lack of significant pairwise differences in *post hoc* analyses, suggests that while there are overall differences between countries and age groups, these differences may not be pronounced enough at the pairwise level. This could be due to the conservative nature of the post hoc test (51), small effect sizes (52), or variability within groups (53).

The disparities observed in this study emphasize the need for targeted public health strategies to enhance HPF while accounting for factors such as BMI and contextual influences. Countries with lower performance, such as Egypt, would benefit greatly from national-level policies aimed at improving PE programs and increasing access to organized sports. Early interventions focused on fitness development, particularly in areas such as sprint speed, abdominal strength, and endurance, are critical for reducing the risk of chronic diseases later in life (23, 54). Educational institutions play a pivotal role in implementing these interventions, as inclusive and culturally relevant PE programs can help address barriers such as gender disparities and unequal access to physical activity opportunities (55). The success of countries like Spain demonstrates the effectiveness of well-structured, evidence-based programs in fostering physical development and promoting long-term health. By adopting similar strategies tailored to their specific needs, countries with lower HPF levels, such as Egypt, can work toward achieving more equitable health outcomes for their youth populations.

The cross-country differences in HPF observed in this study likely stem from variations in PE curricula and their implementation, even after accounting for BMI. Structured PE programs that emphasize sports and fitness from an early age contribute to enhanced physical development in sprint speed, coordination, endurance, and strength (11–14). The consistent advantage in HPF observed in European Mediterranean countries and Lebanon highlights the effectiveness of these programs. For example, Italy’s PE curriculum, which prioritizes general fitness and motor coordination over competitive sports fostering motor skill development without the intensity of sport-specific training (15). In contrast, Egypt faces additional challenges, such as inadequate infrastructure and limited resources, which contribute to its lower performance in several fitness components, including endurance and strength, particularly among girls (19).

These disparities underscore the importance of addressing both systemic and individual-level challenges to improve HPF in children. Barriers such as poor-quality PE classes, a limited variety of activities, insufficient equipment, and inadequate teacher training and commitment have been consistently reported across different contexts (56, 57). Furthermore, low levels of student participation, often linked to a lack of interest and engagement, pose significant obstacles to achieving the desired outcomes of school-based PE programs (58). However, facilitators such as increasing the diversity of PE activities, improving teacher training (particularly for temporary staff), and fostering peer and parental support have been identified as critical strategies to enhance active participation and improve fitness levels (26, 59–62). Implementing these evidence-based interventions could help address the disparities in physical fitness among children and enhance the effectiveness of school-based programs in promoting HPF and reducing inequalities. By targeting improvements in PE programs and addressing these barriers, balanced fitness development and equitable health outcomes can be fostered across diverse educational contexts.

Adapting PE curricula to address country-specific strengths and weaknesses can significantly enhance HPF across diverse cultural contexts. For instance, in countries like Egypt, where children demonstrate lower performance in cardiovascular endurance and strength, PE programs focusing on endurance-building and strength-oriented activities could be particularly beneficial. Furthermore, implementing age-appropriate fitness goals that align with observed age-related improvements will also optimize fitness outcomes. These recommendations align with frameworks such as the Social Ecological Model and Life Course Theory, which highlight the impact of cultural, social, and environmental factors on physical activity and fitness outcomes (3, 22). Research has consistently shown that HPF in children varies significantly across regions, influenced by societal norms, family dynamics, and educational contexts (24–26). Comparative studies like those by Luz et al. (27) and Bardid et al. (28), reveal how motor competence and fitness levels differ due to cultural practices and PE infrastructure. Cross-cultural research is essential for understanding these contextual influences and supporting public health strategies that ensure equitable access to fitness opportunities across diverse cultural settings (29). By developing culturally and developmentally tailored fitness programs, public health officials and educators can foster balanced fitness development and reduce the long-term health risks associated with physical inactivity.

While this study provides valuable insights into HPF across multiple Mediterranean countries, several limitations must be acknowledged. First, the sample size and regional coverage were limited, potentially affecting the generalizability of the findings. Future research should aim to include larger, more representative samples from a wider range of regions within each country to capture a more comprehensive range of fitness outcomes. Second, the study did not account for biological maturation, a key factor influencing physical development, particularly for strength and speed-related components. Variations in maturation rates may explain some of the observed differences in physical fitness outcomes. Therefore, future studies should incorporate measures of biological maturation to provide a more accurate understanding of developmental trajectories for different fitness components.

Additionally, socio-economic factors, dietary habits, and motivational aspects, which are known to significantly influence physical fitness, were not considered in this study. These variables may differ across cultural contexts and contribute to variations in fitness outcomes. Future research should explore the role of these factors to offer a more holistic view of the determinants of HPF among children. Finally, longitudinal studies are needed to track changes in fitness levels over time. These studies would provide valuable insights into how PE interventions or lifestyle changes impact fitness development in diverse cultural contexts. Addressing these limitations will enable the design of more culturally relevant, evidence-based interventions to promote balanced fitness development across populations.

## 5 Conclusion

This study highlights the significant influence of cultural, educational, and environmental factors on HPF components among children in five Mediterranean countries. Regions with well-established PE programs and higher levels of sports participation demonstrated stronger performance in various fitness components, underscoring the importance of structured PE and accessible sports opportunities. In contrast, areas with lower fitness performance emphasize the need for policy-level interventions to enhance access to quality PE and sports programs. These cross-cultural comparisons offer valuable insights for developing targeted public health strategies that promote equitable fitness opportunities and support healthy lifestyles for children across diverse cultural settings. By addressing these disparities, policymakers and educators can implement tailored approaches that encourage balanced fitness development and foster long-term health benefits at the population level.

## Data Availability

The raw data supporting the conclusions of this article will be made available by the authors, without undue reservation.
